# Acute kidney injury secondary to crystalline cryoglobulinemic glomerulonephritis type I: a case report and literature review

**DOI:** 10.3389/fmed.2026.1667211

**Published:** 2026-09-03

**Authors:** Aichun Liu, Yina Wang, Yu Yan, Shuying Zheng, Bao Dong, Meishun Cai, Xin Li, Chunying Shao, Li Zuo

**Affiliations:** 1 Department of Nephrology, Peking University People’s Hospital, Beijing, China; 2 Electron Microscope Laboratory, Peking University People’s Hospital, Beijing, China

**Keywords:** acute kidney injury, crystalline cryoglobulinemic glomerulonephritis type I, monoclonal immunoglobulin, pseudothrombi, type I cryoglobulins

## Abstract

**Background:**

Type I cryoglobulinemia consists of a single monoclonal immunoglobulin and can induce small-vessel vasculitis with renal involvement. Cryoglobulinemic glomerulonephritis is a rare manifestation, pathologically characterized by membranoproliferative glomerulonephritis (MPGN). In this report, we present a case of type I cryoglobulinemic renal injury characterized by crystalline cryoglobulinemic glomerulonephritis type I. Our objective is to provide a detailed insight into the presentation, underlying diseases, diagnosis, treatment, and outcomes of crystalline cryoglobulinemic glomerulonephritis type I.**Case presentation:** A 38-year-old man presented with acute kidney injury (AKI) without any other systemic symptoms. A kidney biopsy confirmed the presence of abundant pseudothrombi within the glomerular capillary lumens, with no significant proliferative lesions observed. Immunofluorescence microscopy (IF) demonstrated strong, diffuse, homogeneous staining for IgG_1_ and Lambda light chain. Electron microscopy revealed a microtubular substructure characteristic of nephropathy. Following the initial course of rituximab, plasma exchange (PE), and methylprednisolone, the treatment yielded successful results. However, renal dysfunction recurred 18 months later, prompting a second renal biopsy, which exhibited findings similar to the first. Additionally, bone marrow biopsy identified an aberrant monotypic plasma cell clone. Subsequently, daratumumab therapy was initiated, leading to a recovery in kidney function.

**Conclusion:**

In nephropathies associated with crystalline cryoglobulinemic glomerulonephritis type I, renal and bone marrow biopsies play a crucial role in establishing the diagnosis and determining the optimal therapeutic approach. It is essential to consider primary diseases prior to selecting the initial management strategy.

## Introduction

Cryoglobulins are immunoglobulins that precipitate at temperatures below 37 °C and redissolve upon warming. Cryoglobulinemia is defined by the persistent detection of cryoglobulins in serum and may present without symptoms. Major clinical manifestations include cutaneous lesions (purpura, necrotic ulcers), arthropathy, peripheral neuropathy, and renal injury (membranoproliferative glomerulonephritis) (1, 2). Cryoglobulinemic glomerulonephritis is observed in approximately 30% of cases of cryoglobulinemia (3). It represents a histopathological entity that can manifest with various proliferative or sclerosing patterns on light microscopy (LM), with membranoproliferative glomerulonephritis (MPGN) being the most prevalent histological pattern, identified in 70%–90% of cases (4). Additionally, it may be associated with a minor degree of microthrombosis. Cases characterized by pseudothrombosis with microtubular structures by electron microscopy as the primary pathological lesion are rare and defined as crystalline cryoglobulinemic glomerulonephritis type I (5). In this report, we present a case of crystalline cryoglobulinemic glomerulonephritis type I characterized by intracapillary pseudothrombi without significant proliferative lesions. We summarize the cases in the literature that meet the diagnostic criteria for crystalline cryoglobulinemic glomerulonephritis type I, aiming to enhance the understanding and clinical management of this pathological pattern.

### Case

A 38-year-old man with no prior history of renal disease was referred to our nephrology department due to oliguric acute kidney injury (AKI). On physical examination, the patient’s temperature was 36.5 °C, blood pressure was 153/97 mmHg, body weight was 70 kg and height was 175 cm. Apart from mild bilateral lower extremity edema, there were no other positive findings. Laboratory test results indicated a hemoglobin level of 84 g/L, serum albumin of 34 g/L, blood urea nitrogen (BUN) of 26.1 mmol/L, and serum creatinine (Scr) of 10.6 mg/dL. Urinalysis demonstrated proteinuria (+++), occult blood (+++), and a 24-h urine protein level of 2.2 g/d. Initial autoimmune workup yielded the following results: serum IgG, 710 mg/dL (reference range: 720–1680 mg/dL); serum IgA, 131 mg/dL (reference range: 82–453 mg/dL); and serum IgM, 60.8 mg/dL (reference range: 46–304 mg/dL). Complement C3 was measured at 0.494 G/L (reference range: 0.9–1.8 G/L) and complement C4 at 0.116 G/L (reference range: 0.1–0.4 G/L). Anti-nuclear antibody (ANA) was detected at a titer of 1:40 using indirect immunofluorescence (laboratory cutoff = 1:40; titers >1:40 are regarded as clinically significant). Additionally, anti-myeloperoxidase (MPO)-ANCA was 199.31 RU/mL by Enzyme-Linked Immunosorbent Assay (reference range: 0–20 RU/mL), and anti-proteinase 3(PR3) -ANCA was 307.6 RU/mL by Enzyme-Linked Immunosorbent Assay (reference range: 0–20 RU/mL). ANCA retesting by indirect immunofluorescence, performed at an external laboratory, yielded negative results. Protein electrophoresis revealed a weak monoclonal spike of the IgG-Lambda type in both serum and urine samples. The serum free light chain (FLC) Lambda measured 32.5 mg/L (reference range: 5.7–26.3 mg/L), while the FLC Kappa chain was 15.4 mg/L (reference range: 3.3–19.4 mg/L). The serum FLC Lambda/Kappa ratio was calculated at 0.47 (reference range: 0.26–1.65). Notably, a serum cryoglobulins test returned a negative result. A bone marrow biopsy did not demonstrate any morphologic features indicative of involvement by a lymphoproliferative or plasma cell proliferative disorder. Immunophenotyping showed that CD38+CD138+CD19+ plasma cells constituted 0.02% of the total cells, and no aberrant phenotype was detected. To elucidate the underlying causes of proteinuria, hematuria, and AKI, a percutaneous renal biopsy was subsequently performed.

**FIGURE 1:**
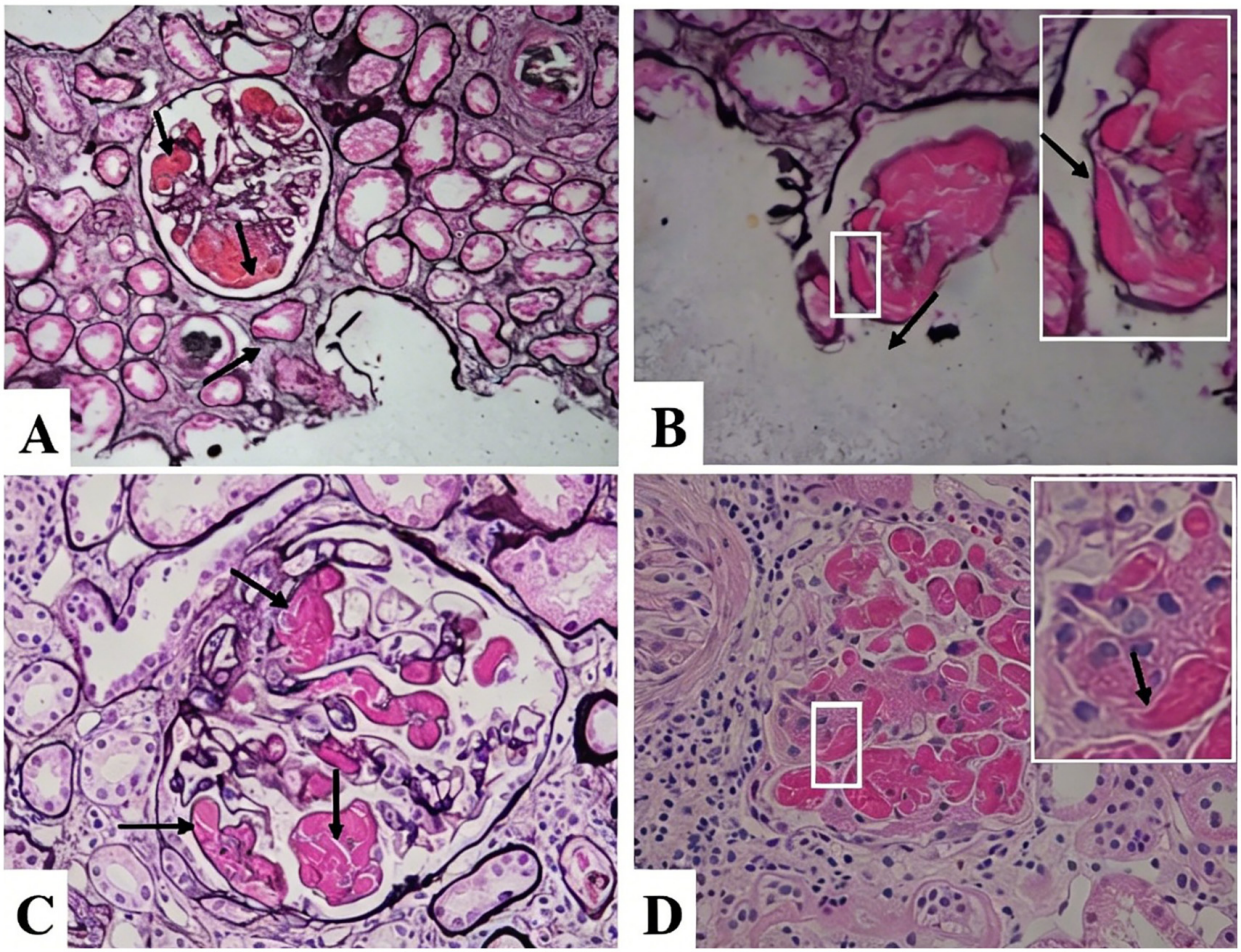
Light microscopic images **(A–D**, ×400**)**. **(A,B)** Are from the first renal biopsy. **(C,D)** Are from the second renal biopsy. Unstructured embolus-like substances lacking cellular components were observed in the glomerular capillary **(**arrows in **A–D)**. Characterized by the presence of at least one straight edge, these inclusions are termed glomerular crystals. The total glomeruli exhibited mesangial and mild endocapillary proliferation. No crescent was formed.

**FIGURE 2:**
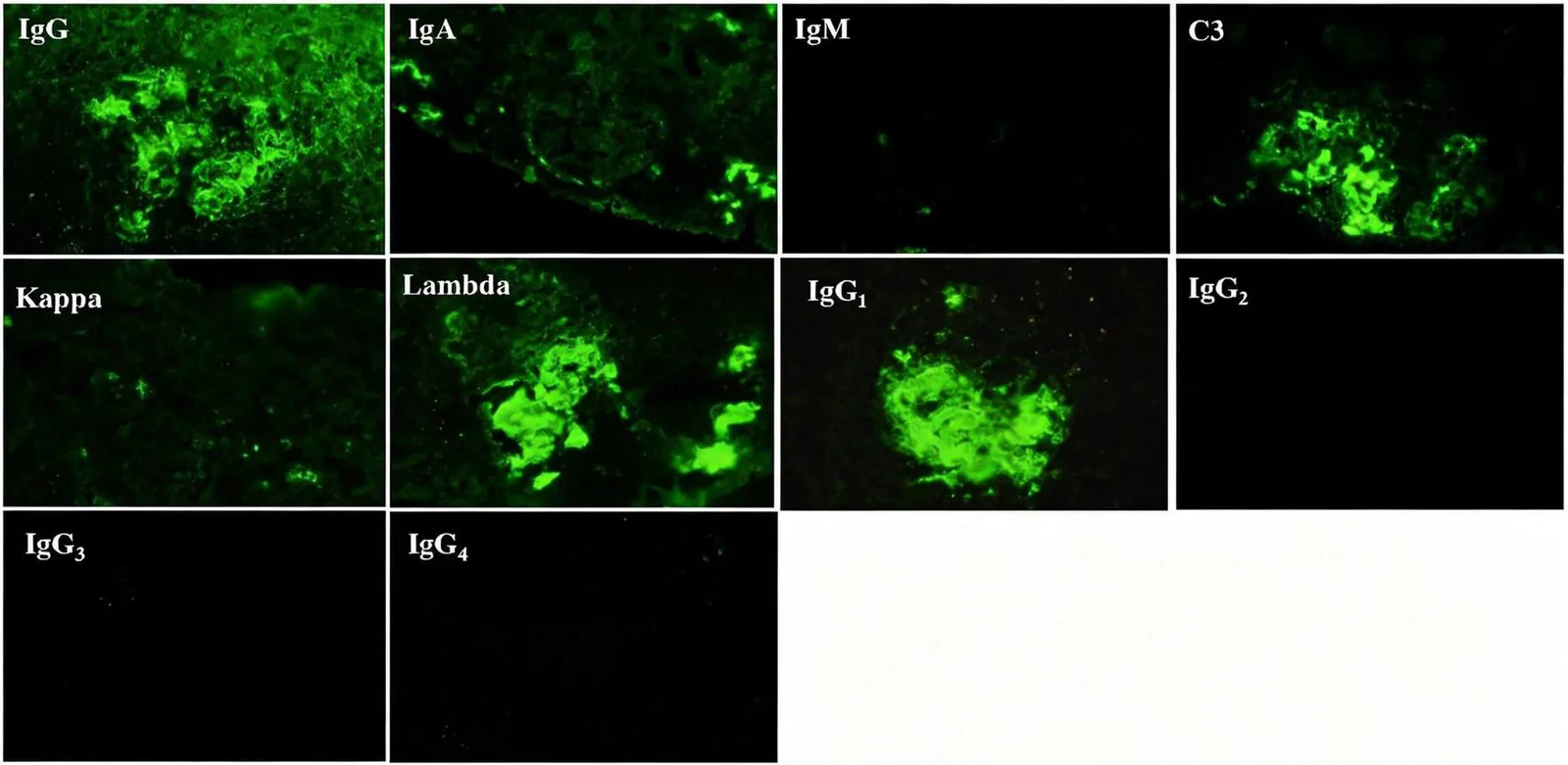
Immunofluorescence. Glomerular intracapillary immunoglobulin pseudo thrombi show staining for immunoglobulin G (IgG) and Lambda light chain and are negative for Kappa, IgM, IgA, and C1q. There is segmental glomerular capillary loop staining for C3. For IgG subtype, the thrombi were only strongly reactive with IgG_1_, negative with IgG_2_, IgG_3_ and IgG_4_.

**FIGURE 3:**
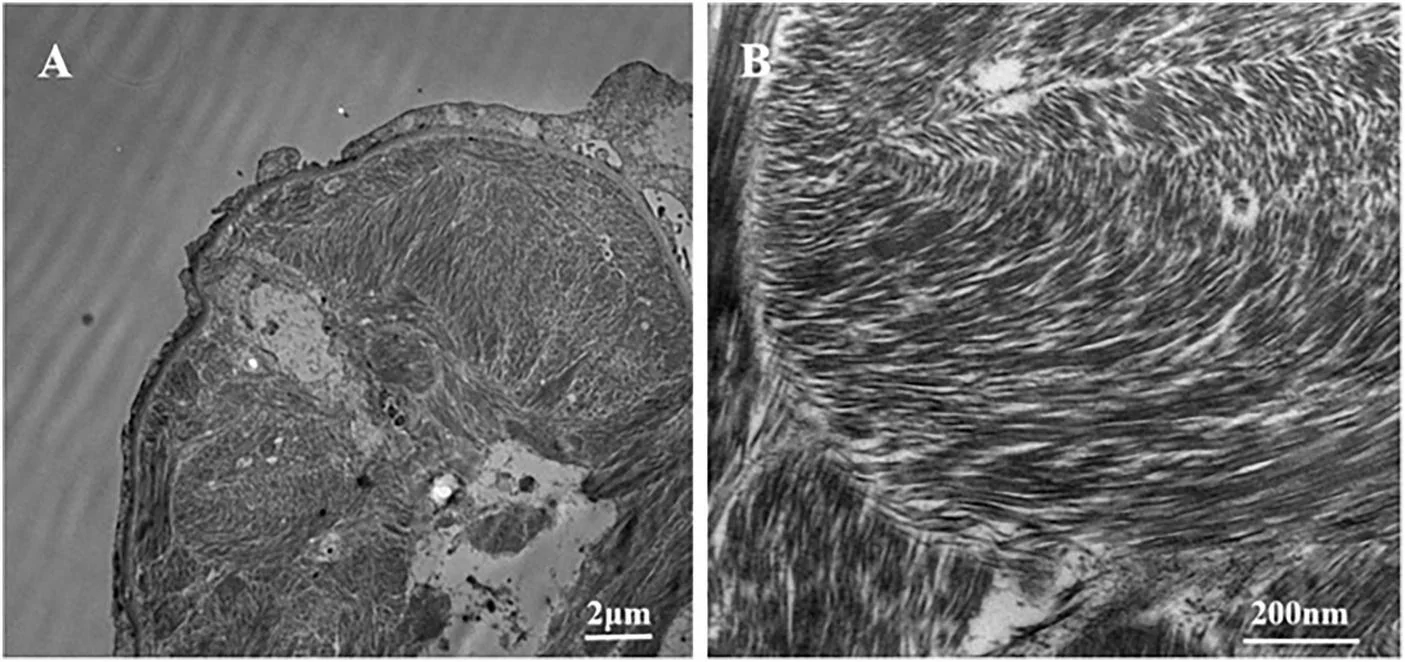
Electron microscopic images. **(A)** Intracapillary immunoglobulin pseudothrombi are present in glomeruli; **(B)** on higher magnification, these deposits show a microtubular structure typical of cryoglobulins of approximately 20–30 nm.

The patient’s kidney biopsy revealed a significant presence of glomerular capillary pseudothrombi that stained positively for IgG_1_ and Lambda using immunofluorescence (IF). Light microscopy (LM) demonstrated a considerable number of pseudothrombi within the glomerular capillaries. The embolus-like substances found in the glomeruli were fuchsin positive when subjected to Masson trichrome staining, while Congo red staining yielded negative results. The glomeruli displayed mesangial and mild endocapillary proliferation, with no thickening observed in the arterial endothelium (Figures 1A, B). IF examination indicated deposition of IgG in the areas of embolus-like substances, whereas IgM, IgA, and C1q were negative in the glomerular capillary thrombi. Staining for IgG subtypes revealed positivity for IgG_1_ only, with negative results for IgG_2_, IgG_3_, and IgG_4_. Regarding light chains, Lambda light chain was positive in the thrombi, while Kappa light chain was negative. No extraglomerular deposits were detected (Figure 2). Electron microscopy analysis of the intracapillary pseudothrombi exhibited a substantial microtubular structure measuring 20–30 nm, characteristic of cryoglobulins (Figure 3).

Based on the pathological findings from the renal biopsy, the patient was ultimately diagnosed with crystalline cryoglobulinemic glomerulonephritis type I (IgG_1_-Lambda type) affecting the glomeruli. Due to the rapid kidney injury caused by paraproteinemia, plasma exchange (PE) was incorporated into the 3-day methylprednisolone (mPSL) pulse therapy (500 mg/day), which resulted in a swift response. Plasma exchange was performed every other day for a total of seven sessions. The replacement fluid consisted of 2000 ml of fresh frozen plasma, 500 ml of crystalloid solution (Ringer’s solution), and 500 ml of albumin saline solution (40 g/L), resulting in a total exchange volume of 3000 ml per session. Hemodialysis was subsequently discontinued on day 10. Following the pulse therapy, prednisolone was administered at a dosage of 40 mg/day. Given that B-cell lymphocytes are responsible for the production of the monoclonal IgG_1_-Lambda, rituximab (RTX), a monoclonal antibody targeting the CD20 antigen present on B lymphocytes, was utilized to achieve complete B-cell depletion. The patient demonstrated a significant response to the treatment, with urine output improving to the normal range and a downward trend in creatinine levels to 1.5 mg/dL. M protein could not be detected in the serum or urine via immunoelectrophoresis, and complement levels C3 and C4 returned to normal. However, autoantibodies remained notably positive, including anti-MPO-ANCA, and anti-PR3-ANCA.

**FIGURE 4:**
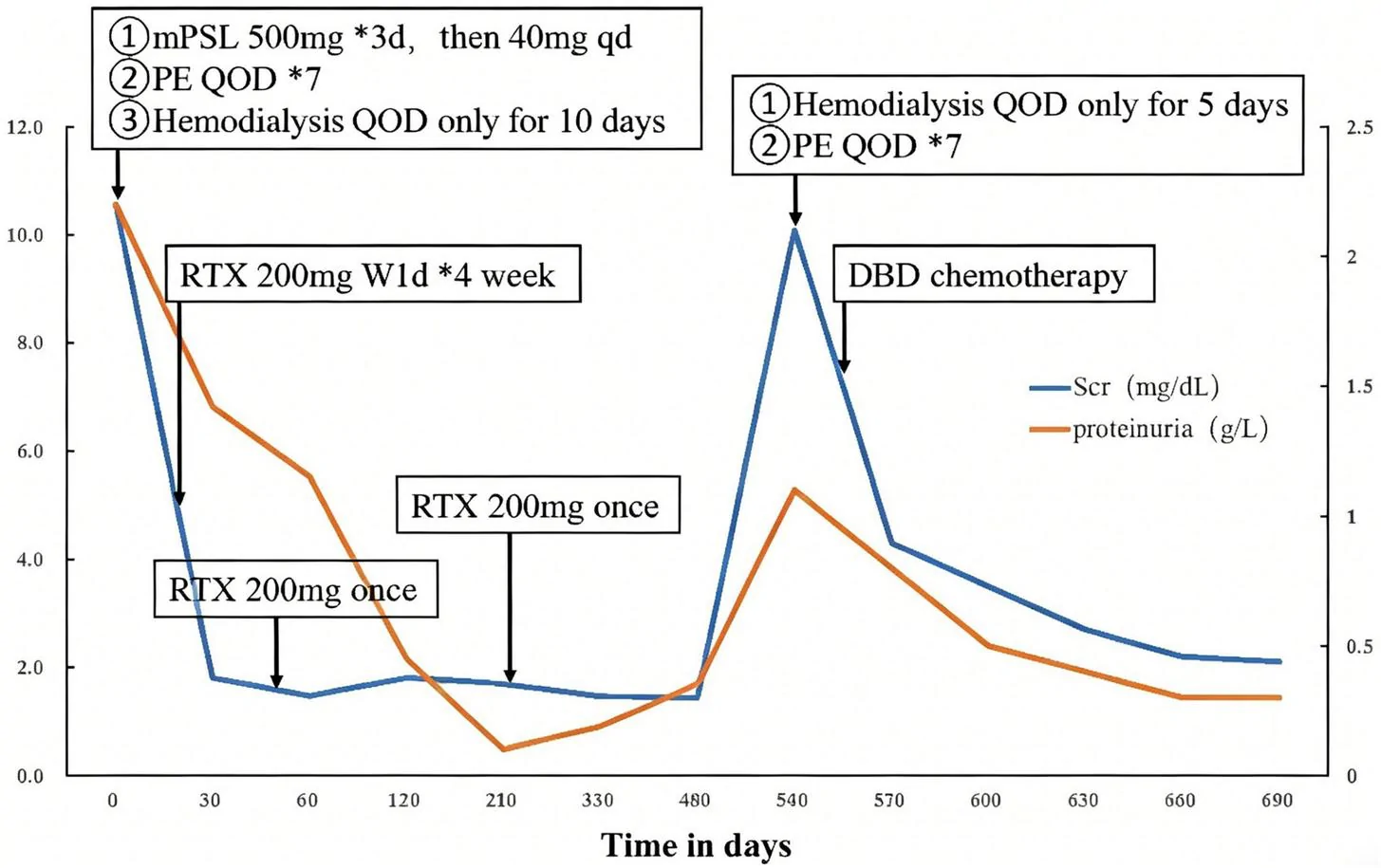
The clinical treatment and prognosis of this patient. mPSL, methylprednisolone; PE, plasma exchange; RTX, rituximab; DBD, daratumumab (Dara), bortezomib, and dexamethasone (Dex).

The patient, treated with rituximab, exhibited complete B-cell depletion. However, 18 months later, upon reducing the prednisone (PSL) dosage to 5 mg/day, serum creatinine levels deteriorated to 10.1 mg/dL, necessitating readmission. Monoclonal IgG-Lambda reappeared in serum and urine immunoelectrophoresis. Other laboratory investigations during the second admission were consistent with those from the first admission. The second bone marrow biopsy revealed abnormal findings. The bone marrow smear showed that plasma cells accounted for 1.5% of all nucleated cells. Immunophenotyping indicated that plasma cells constituted 0.12% of the total cell population, of which 84.24% were aberrant clonal plasma cells expressing CD45dim, CD38, CD138, cLambda, CD27, CD9, and CXCR4. No CD20-positive B cells were detected. Consequently, a second renal biopsy was performed to evaluate the cause of renal re-injury. The kidney biopsy demonstrated glomeruli obstructed by immunoglobulin pseudothrombi, which stained positively for IgG_1_ and Lambda by immunofluorescence, resembling the findings of the first renal biopsy (Figures 1C, D). The patient was referred to the hematology department due to the identification of clonal proliferation of plasma cells. He subsequently received plasma exchange using the same frequency and regimen as the initial treatment, combined with a daratumumab-containing targeted therapy for a total of five chemotherapy cycles. The specific dosing schedule is described as follows. In the first cycle, the DBD regimen was administered: dexamethasone 20 mg on days 1, 8, 15, 21, and 28; bortezomib 2 mg on days 1, 8, 15, 21, and 28; and daratumumab 800 mg on days 1, 15, 21, and 28. The second and third cycles followed the same DBD regimen, with dexamethasone 20 mg on days 1, 8, 15, and 21; bortezomib 2 mg on days 1, 8, 15, and 21; and daratumumab 800 mg on days 1 and 15. The fourth cycle also adopted the DBD regimen, with the same dosing schedule for dexamethasone and bortezomib as in the previous cycles, while daratumumab was administered only on day 1. This regimen reduced the patient’s serum creatinine level from 10.1 mg/dL to 2.1 mg/dL, and no adverse reactions were observed. The time course of laboratory studies, and treatment is presented in Figure 4.

## Discussion

Herein, we present a rare case of IgG_1_-Lambda type I cryoglobulinemic glomerulonephritis resulting in intracapillary pseudothrombi, a histopathological entity currently classified as type I crystalline cryoglobulinemic glomerulonephritis. For the first time, we conducted a subtype analysis of IgG. The patient experienced two episodes of AKI, both attributed to thrombus obstruction in the glomerular capillary lumen. Kidney biopsies and bone marrow biopsies confirmed the renal pathology and the progression of the underlying hematological disease. This case exhibited a favorable response to PE and targeted monoclonal therapy, leading to a rapid improvement in renal function.

At the same time, we conducted a literature search using the keywords “monoclonal immunoglobulin,” “type I cryoglobulinemias,” “pseudothrombi,” “monoclonal gammopathy of renal significance” and “kidney injury” in PubMed, Medline, and Web of Science, focusing on relevant publications from the establishment of the database until Dec, 2024. We reviewed a total of 23 cases in the literature involving pseudothrombosis within the glomerular capillary lumen. Monoclonal immunoglobulins (MIgs), particularly IgM, are readily deposited in the glomeruli, leading to the formation of thrombosis-like substances. However, not all thrombosis-like substances are classified as cryoglobulin. Thrombi characterized by microtubule-like, fibrous, or granular substructures, as observed under electron microscopy, are identified as cryoglobulins. According to this newly published consensus, cryoglobulinemic glomerulonephritis is classified into two major categories. Specifically, “crystalline cryoglobulinemic glomerulonephritis type I” is characterized by the presence of glomerular crystals, which can be either intracellular (within macrophages or endothelial cells) or extracellular (intraluminal, subendothelial, or mesangial). Supportive histological features include intraluminal pseudothrombi and granular electron-dense deposits observed via electron microscopy (EM). Consequently, we excluded 20 patients who did not meet this definition. Table 1 summarizes the clinical and pathological characteristics of all four identified cases (including our patient) that fulfilled the diagnostic criteria for type I crystalline cryoglobulinemic glomerulonephritis. The average age of the patients was 50.5 ± 10.6 years. The clinical manifestations of renal damage primarily included AKI, accompanied by proteinuria and hematuria.

**TABLE 1:** Features of the patients reported in the literature and the present case.

Author/year (country)	Age/ gender	Renal manifestation			MIgs	Cryoglobulins	LM			IF		EM	Bone marrow biopsy	Treatment	Renal outcome
		Urine protein, g/d	Cr, mg/dL	AKI			Proliferative lesions	Pseudo-thrombi in glomeruli	Tubuloin terstitial lesions	High chain	Light chain				
Gupta et al. (6), (USA)	63/M	>3	4.08	Y	IgAκ	Negative	MPGN	+	−	IgA	Kappa	Lattice-like	MGUS+ CLL	Symptomatic treatment	MHD
Okazaki et al. (1), (Japan)	47/M	0.28 g/d	4.55	Y	IgGκ	NA	−	+	MIg cast	IgG	Kappa	Cylinder-like, 60 nm	MGUS	PE, chemotherapy	Remission
Hamadah et al. (7), (USA)	54/M	0.5 g/d	7.5	Y	−	Positive	MPGN	+	−	IgG	Kappa	Microtubular	Normal	PE, chemotherapy	Remission
Present case (China)	38/M	2.2	10.6	Y	IgGλ	Negative	−	+	−	IgG_1_	Lambda	Microtubular, 20–30 nm	Normal	PE, chemotherapy	Remission

MGUS, monoclonal gammopathy of undetermined significance; MIgs, monoclonal immunoglobulins; MPGN, membranoproliferative glomerulonephritis; LM, light microscope; IF, immunofluorescence; EM, electron microscopy; MHD, maintenance hemodialysis; NA, not available; PE, plasma exchange.

Renal injury demonstrates heterogeneous pathological features. Two patients presented with isolated cryoglobulin pseudothrombi occluding capillary lumens, one of whom harbored protein casts within tubular lumens. Two other patients showed concurrent MPGN. Some studies suggest that these observations may be attributed to differing levels of disease activity or may represent different stages of the same disease. Furthermore, “isolated pseudothrombosis” may not constitute a separate renal manifestation but rather an early form of cryoglobulin-related renal injury (8, 9). If this initial stage is neither detected nor treated promptly, MPGN may develop as the local immune response becomes activated.

Various types of cryoglobulin can lead to pseudothrombosis. Three cases of IgG type I cryoglobulins, and one case of IgA type I cryoglobulins have been documented. Notably, our patient represents the first case of pseudothrombosis caused by IgG_1_ type I cryoglobulin within the capillary lumen. The aggregation ability of different immunoglobulin (Ig) heavy and light chains is not determined solely by specific sequence factors; rather, it results from a combination of multiple factors (10). Research indicates that two-thirds of heavy chain deposition disease (HCDD) cases secrete truncated monoclonal heavy chains with CH1 deletion (11). Furthermore, a given protein may exhibit more than one critical intermediate conformation during the aggregation pathway, and these distinct conformations may lead to different types of deposits (12). The replacement of hydrophobic residues, N-glycosylation, and an increase in the isoelectric point of MIgs contribute to the transformation from a soluble form to tissue deposition, ultimately resulting in glomerular damage (13–15). Furthermore, the deposition of MIgs is influenced by the interplay between specific somatic mutations and the surrounding conditions of the protein (16). We speculate that the aggregation and precipitation of IgG_1_-Lambda type I cryoglobulin in this patient result from a combination of the intrinsic characteristics of the sequence and external conditions. In the future, laser microdissection coupled with mass spectrometry analysis will be feasible for determining the specific structure of MIg deposited in the kidney (17).

In this study, only one patient tested positive for blood cryoglobulins, while two patients tested negative, and one case was not described. Nevertheless, the renal pathology of all patients clearly confirmed the diagnosis of cryoglobulinemic glomerulonephritis. It is not uncommon to observe classic cases of cryoglobulinemic glomerulonephritis even in the absence of positive serum cryoglobulins. This discrepancy may arise from improper serum sample collection or handling, cyclical variations in the levels of circulating cryoglobulins, and/or their physicochemical properties that promote precipitation in the glomerulus, leading to relative dilution in the serum (11). Although cryoglobulins were not detected in some patients, MIgs were identifiable in blood and/or urine immunofixation in the majority of cases (3 out of 4, 75%). This phenomenon is likely attributable to the low concentration of MIgs in the serum, which fails to aggregate into cryoglobulins (18). Initially, when our case presented with AKI, the blood MIgs concentration was only weakly positive, measuring 0.1 g/L. Furthermore, the blood cryoglobulin test results were negative, and the bone marrow biopsy appeared normal. It is hypothesized that an underlying factor, such as an infection, may have stimulated lymphoid B cells to produce a small quantity of monoclonal immune proteins. These proteins circulate in the bloodstream and are filtered through the glomeruli, leading to an increased local concentration, deposition, and accumulation in the kidneys, ultimately resulting in the formation of cryoglobulins.

The primary mechanism of monoclonal gammopathy-associated renal lesions (MGRS) involves abnormal deposition or pathological activity of M-proteins within the kidneys. M-proteins may originate from pre-existing benign or pre-neoplastic hematological disorders, such as monoclonal gammopathy of undetermined significance (MGUS) or smoldering multiple myeloma. Nevertheless, a subset of patients with histologically confirmed MGRS exhibit undetectable MIgs in serum and urine, presumably owing to M-protein concentrations below the analytical detection threshold. Owing to abundant renal blood flow, glomerular filtration, and charge-dependent interactions, MIgs become highly concentrated within glomeruli, where they crystallize or form granular deposits. These deposits activate complement cascades and inflammatory signaling pathways, thereby eliciting renal injury. Renal manifestations may precede overt hematological malignancy. The detection rate of pathogenic clones varies across distinct MGRS subtypes. For instance, in two studies enrolling patients with light-chain deposition disease, bone marrow biopsy identified clonal plasma cells in 65% to nearly 100% of cases (19). By contrast, patients with proliferative glomerulonephritis with monoclonal immunoglobulin deposits (PGNMID) display a markedly lower clonal detection rate of 25%–30% (20, 21). Pooled analyses of major MGRS case series indicate that clonal populations remain undetectable in approximately 40% of affected patients (22).

In terms of treatment, all three patients received chemotherapy and PE at the early stage of diagnosis, which resulted in significant relief of renal involvement. One patient was unable to tolerate chemotherapy due to a poor overall condition and subsequently required maintenance dialysis. Glomerular diseases associated with MIgs deposition should be considered paraneoplastic glomerulopathies, and a bone marrow biopsy is essential to identify the source of MIgs, which is crucial for guiding treatment (7). Glucocorticoids can suppress the inflammatory response, while PE can directly eliminate circulating IgG complexes; however, PE does not address the underlying disease, leading to a potential rapid increase in circulating IgG levels subsequently (23). Therefore, targeting the pathogenic clone with chemotherapy is crucial (1, 7). In our case, the disease progression was categorized into two stages. In the first stage, the bone marrow biopsy appeared normal, leading us to conclude that B lymphocytes were producing monoclonal IgG-Lambda. Consequently, the patient was treated with rituximab to achieve B-cell depletion, which resulted in the anticipated improvement in renal function. Notably, renal monoclonal gammopathy may appear prior to bone marrow involvement. Yet we have no adequate evidence to validate this hypothesis. In the second stage, following complete B-cell depletion, serum creatinine levels deteriorated again, monoclonal IgG-Lambda re-emerged, and the bone marrow biopsy revealed the presence of abnormal clonal plasma cells. The bone marrow contains only a small number of clonal plasma cells, below the threshold for myeloma. However, pathogenic monoclonal immunoglobulins from these cells cause acute kidney injury. Active treatment is therefore necessary. The patient subsequently underwent therapy with Daratumumab, which once again improved his renal function. Daratumumab is a humanized IgG_1_ Kappa monoclonal antibody against CD38. It specifically binds to the CD38 epitope on the cell surface and induces cytolysis and plasma cell death through multiple mechanisms (24–26). Dara significantly improves the response rate and progression-free survival in newly diagnosed and high-risk patients with multiple myeloma (MM) (27). In recent years, daratumumab has demonstrated breakthrough efficacy in the treatment of plasma cell clone-associated MGRS, including AL amyloidosis (28), light chain proximal tubulopathy (29), and PGNMID (30). Our patient represents the first case treated with Dara for type I cryoglobulin-mediated renal injury, achieving partial renal remission. Accordingly, early evaluation of CD38 expression is strongly recommended for MGRS caused by clonal plasma cell disorders. Under multidisciplinary collaboration between the hematology and nephrology departments, early incorporation of Dara into the therapeutic regimen should be considered to achieve profound hematological remission, which serves as the prerequisite for the recovery of organ function. This observation also suggests that the source of MIgs may vary at different stages within the same patient. Furthermore, timely renal and bone marrow biopsies can facilitate the diagnosis of the underlying cause and the implementation of targeted treatment.

In conclusion, crystalline cryoglobulinemic glomerulonephritis type I can be driven by various types of MIgs forming pseudothrombi within the glomerular capillary lumen, with IgG_1_ type I cryoglobulin being exceedingly rare. Clinically, AKI and proteinuria are the primary manifestations. Timely kidney and bone marrow biopsies are essential for confirming the diagnosis and adjusting treatment accordingly. The combination of PE with targeted chemotherapy can significantly enhance renal outcomes.

## Data Availability

The raw data supporting the conclusions of this article will be made available by the authors, without undue reservation.
